# Deep brain stimulation and responsiveness of the Persian version of Parkinson’s disease questionnaire with 39-items

**Published:** 2014-10-06

**Authors:** Gholam Ali Shahidi, Zeinab Ghaempanah, Yasaman Khalili, Marzieh Nojomi

**Affiliations:** 1 Department of Neurology, School of Medicine, Rasoule-Akram Hospital, Iran University of Medical Sciences, Tehran, Iran; 2 Department of Psychology, School of Humanities, Tarbiat-Modares University, Tehran, Iran; 3 Department of Community Medicine, School of Medicine, Iran University of Medical Sciences, Tehran, Iran; 4 Department of Community Medicine, School of Medicine, Preventive Medicine Research Center, Iran University of Medical Sciences, Tehran, Iran

**Keywords:** Deep Brain Stimulation, Parkinson’s Disease Questionnaire with 39-Items, Responsiveness

## Abstract

**Background: **Assessment of quality-of-life (QOF) as an outcome measure after deep brain stimulation (DBS) surgery in patients with Parkinson’s disease (PD) need a valid, reliable and responsive instrument. The aim of the current study was to determine responsiveness of validated Persian version of PD questionnaire with 39-items (PDQ-39) after DBS surgery in patients with PD.

**Methods:** Eleven patients with PD, who were candidate for DBS operation between May 2012 and June 2013 were assessed. PDQ-39 and short-form questionnaire with 36-items (SF-36) were used. To assess responsiveness of PDQ-39 standardized response mean (SRM) was used.

**Results: **Mean age was 51.8 (8.8) and all of the patients, but just one were male (10 patients). Mean duration of the disease was 8.7 (2.1) years. Eight patients were categorized as moderate using Hoehn and Yahr (H and Y) classification. All patients had a better H and Y score compared with the baseline evaluation (3.09 vs. 0.79). The amount of SRM was above 0.70 for all domains means a large responsiveness for PDQ-39.

**Conclusion: **Persian version of PDQ-39 has an acceptable responsiveness and could be used to assess as an outcome measure to evaluate the effect of therapies on PD.

## Introduction

Parkinson’s disease (PD) is a neurological and progressive disorder of central nervous system. The average prevalence of this disease is about 2% in the general population of 65 years and more.^1^ There is not any known neuroprotective or regenerative treatment for this disease.^2^ This disease causes disability and affects on health-related quality-of-life (QOL) of patients in advanced stages substantially.^3^ The main treatment for this disease is using levodopa (L-dopa). This drug causes long-term complications such as motor fluctuations and dyskinesias.^4^ Some papers have reported marked motor benefits of deep brain stimulation (DBS) in patients with PD.^5^^,^^6^ This surgical procedure is a reversible “non-lesioning” treatment using electrodes implanted into the brain. This method can improve motor functions by 41% after 12 months. Almost one-third of patients with PD under DBS have shown a substantial improvement in QOL.^7^ It has been shown using this surgical method levodopa daily use can be reduced by 48% and 50% at 1 and 4 years respectively.^8^

QOL is an important outcome measure in the evaluation of any intervention in chronic disease. Majority of trials on PD have evaluated QOL as an outcome measures.^9^^-^^12^ Assessment of the effectiveness of interventions on the patient’s concerns needs a valid and responsive instrument. The subjective assessment of the effect of the PD on QOL of patients as a patient-oriented variable has become possible by some generic and specific instruments. One of the valid and reliable instruments to assess QOL of patients with PD is the PD questionnaire with 39-items (PDQ-39). This questionnaire was developed by Jenkinson et al.^13^ and Peto et al.^14^ This instrument has 39-items included eight domains and has been adapted into more than 40 different cultures and languages.^15^ It has been shown the Persian version of PDQ-39 is valid and reliable to assess the QOL of patients with PD.^16^

It is widely discussed that outcome measures in clinical trials should be reliable, valid and responsive.^17^ It is the ability of a measure to change over a pre-specified time.

The aim of the current study is to evaluate the responsiveness of PDQ-39 in patients with PD who take DBS in a prospective study.

## Materials and Methods

Eleven patients with PD who were candidate for DBS operation between May 2012 and June 2013 at the Neurology Department of Rasoule Akram Teaching Hospital in Tehran, Iran were recruited. The inclusion criteria were fulfilling the clinical diagnosis criteria of the United Kingdom brain-bank for PD.^18^

Inclusion criteria were ages <65 years, duration of disease >5 years, disability resulting from either hypokinetic or hyperkinetic motor fluctuations or tremor not adequately controlled with best medical treatment, improvement of motor symptoms >50% with a doubled morning dose of levodopa for each patient.

Exclusion criteria were severe brain atrophy, coagulopathy, active psychosis, major depression with suicidal ideation, substance abuse, poor general medical condition, cardiac pacemaker, and conditions that needed frequent magnetic resonance imaging (MRI) for follow-up. The study was conducted in accordance with the Declaration of Helsinki. Written consent was obtained from all patients and Institutional Review Board of the Iran University of Medical Sciences approved the study.

The subthalamic nucleus (STN) anatomically targeted by 1.5 T MRI using Leksell frame system on both sides. Anatomical targeting was confirmed by intra-operative microelectrode recording (MER) and macroelectrode stimulation. For MER five electrodes were used for each STN to confirm the correct anatomical targeting and to determine the upper and lower anatomo-physiological boundaries of the STN. Points with either the least neurological side effects or best clinical effects during macroelectrode stimulation selected and quadripolar electrodes were implanted on each side.

We used two questionnaires to evaluate the QOL of PD patients. The main questionnaire in order to assess responsiveness was the PDQ-39 questionnaire.^13^^,^^14^ The PDQ-39 contains eight domains: mobility (10 items), activities of daily living (6 items), emotional well-being (6 items), stigma (4 items), social support (3 items), cognitions (4 items), communication (3 items), and bodily discomfort (3 items). Previous research has suggested the Persian version of this questionnaire has a good internal and test-retest reliability, as well as a good construct and face validity.^16^ In this questionnaire, each item scored from 0 to 4 as the best to worst. Each domain score ranges from 0 (the best QOL) to 100 (the worst QOL).

The second questionnaire as a criterion was the short-form questionnaire with 36-items (SF-36) health status survey, which has previously been validated in Persian.^19^ The SF-36 contains eight subscales measuring aspects of physical and mental health. Each dimension is reported on a scale of 0-100 with a higher score reflected the better QOL. Other variables included demographic variables (age and gender). Clinical variables, including disease duration and severity of disease were also collected. Disease severity and clinical evaluation of the patients was assessed by using Hoehn and Yahr classification.^20^ This classification has five stages. We categorized stages as follows: stages I-II as mild, stage III as moderate, and stages IV-V as severe. Clinical evaluation of the patients was done before surgery and 6 months after that. The same physician in the neurology department had been evaluated the patients clinically across two periods of time. The PDQ-39 and SF-36 questionnaires were offered the day before the surgery and 6 months later to the patients too.

For each non-numeric variable frequency and relative frequency were used. We used mean and standard deviation for numeric variables. Internal consistency of PDQ-39 was reported by Cronbach’s alpha. To assess responsiveness of PDQ-39 standardized response mean (SRM) was used. SRM is a ratio of the observed change and the standard deviation reflecting the variability of change scores $\left(\mathrm{SRM}=\frac{\overset{\text{̅}}{D}x}{\mathrm{SD}\left(\mathrm{Dx}\right)}\right)$. A measure that has a high level of variability in change scores in relation to mean change will have a small SRM value. Values of 0.20, 0.50, and 0.80 or greater have been defined as small, moderate, and large responsiveness respectively.^21^

All domains of PDQ-39 at baseline and 6 months after surgery had a normal distribution using Kolmogorov–Smirnov test (P-value less than 0.050). Results of clinical evaluation were considered as a criterion to identify whether patients had changed over time. We used SF-36 as a second criterion to assess responsiveness of PDQ-39 questionnaire too. We used Spearman non-parametric correlation analysis to assess the association between PDQ-39 and relevant SF-36 domains. Higher score for SF-36 and the lower score for PDQ-39 questionnaire indicate the better QOL. Therefore, negative correlation coefficient between scores of this questionnaire was expected. The SF-36 summary components are computed by three stages. At first, we calculated Z-sores for all eight domains. Second, Z-scores are multiplied by the subscale factors score coefficients for physical component scale (PCS) and mental component scale (MCS) and summed over all eight domains. Finally, t-scores are computed by multiplying PCS and MCS by 10 and adding 50 to the product. Significant level was set at 0.050 level.

## Results

Eleven patients with PD were evaluated to determine the responsiveness of PDQ-39. Demographic characteristics of the patients are illustrated in table 1. Mean age was 51.8 (8.8) and all of the patients, but just one were male (10 patients). Mean duration of the disease was 8.7 (2.1) years. Eight patients were categorized as moderate using H and Y classification. The highest score of PDQ-39 was for activities of daily living (59; ±28.7) and bodily discomfort (40; ±26.3).

Cronbach’s alpha was above 0.7 for six domains of PDQ-39. The least reliability was for cognition and communication (0.50 and 0.43 respectively) (Table 2).

In response to the transition at the 6 month follow-up evaluation, all patients had a better H and Y score compared with the baseline evaluation (3.09 ± 0.53 vs. 0.79 ± 0.64). This difference was statistically significant (P = 0.003).

**Table 1:** Demographic and baseline clinical characteristics of the patients with Parkinson disease

**Characteristics **	**Number (%)**
Number of patients	11
Male, n (%)/female, n (%)	10 (91)/1 (9)
Mean (standard deviation) age, years	51.8 (8.8)
Mean (standard deviation) age at onset, years	43.0 (8.8)
Mean (standard deviation) disease duration, years	8.7 (2.1)
Hoehn and Yahr stage	
Mild	1 (9.1)
Moderate	8 (72.7)
Severe	2 (18.2)
PDQ-39 domains (mean and standard deviation)	
Mobility	25.6 (23.7)
Activities of daily living	59.0 (28.7)
Emotional well being	43.1 (29.3)
Stigma	38.6 (29.8)
Social support	33.7 (33.1)
Cognitions	13.6 (13.6)
Communication	24.2 (17.6)
Bodily discomfort	40.1 (26.3)

Responsiveness or sensitivity to change of the PDQ-39 scales was assessed using SRM. After 6 month follow-up, for all domains of PDQ-39, there was a significant decrease in scores (P < 0.010 for all domains). The amount of SRM was above 0.70 for all domains means a large responsiveness for PDQ-39 (Table 3).

Scores for all domains of SF-36 were higher than before DBS after 6 months follow-up. These changes were statistically significant (P < 0.050) for all domains, but one (role limitation due to physical health). Table 4 illustrates that there is a negative and fine correlation between relevant domains for PDQ-39 and SF-36 6 months after DBS.

**Table 2:** Reliability of Parkinson’s disease questionnaire with 39-items using Cronbach’s alpha

**Domain**	**Number of items**	**Cronbach’s alpha**	**Item total correlation (range)**
Mobility	10	0.94	0.70-0.94
Activities of daily living	6	0.91	0.70-0.91
Emotional well being	6	0.90	0.60-0.95
Stigma	4	0.87	0.81-0.91
Social support	3	0.77	0.78-0.85
Cognition	4	0.50	0.30-0.77
Communication	3	0.43	0.40-0.86
Bodily discomfort	3	0.73	0.40-0.72

**Table 3:** Standardized response mean and significance of change (paired t-test) between baseline and 6 months follow-up for the eight domains of Parkinson’s disease questionnaire with 39-items and short-form questionnaire with 36-items physical component scale and mental component scale

**Domains**	**Standardized response ** **mean**	**Mean t ** **(baseline)**	**Mean t ** **(6 months later)**	**Paired t-test ** **(P)**
PDQ-39 domains				
Mobility	0.84	25.68	08.18	0.019
Activities of daily living	1.75	59.00	12.50	0.001
Emotional well-being	1.10	43.18	15.53	0.004
Stigma	1.07	38.63	07.95	0.005
Social support	0.70	33.71	11.74	0.042
Cognitions	0.95	13.63	05.68	0.011
Communication	1.20	24.24	09.84	0.002
Summary index	1.60	34.30	11.40	0.001
Bodily discomfort	1.27	40.15	11.36	0.002
SF-36 summary scores				
Physical	0.60	64.02	45.65	0.074
Mental	0.17	59.42	54.39	0.587

PDQ-39: Parkinson’s disease questionnaire with 39-items; SF-36: Short-form questionnaire with 36-items

**Table 4:** Spearman correlation coefficients between Parkinson’s disease questionnaire with 39-items and relevant short-form questionnaire with 36-items domains 6 months after deep brain stimulation

**Domain SF-36 and ** **PDQ-39**	**Physical ** **functioning**	**Role limitation due to ** **physical functioning**	**Emotional well ** **being**	**Social ** **functioning**	**Pain**
Mobility	-0.87 (0.001)	-0.57 (0.060)			
Activities daily living	-0.61 (0.040)	-0.35 (0.290)	-	-	-
Emotional well being	-	-	-0.90 (0.001)	-	-
Social support	-	-	-	-0.48 (0.130)	-
Bodily discomfort	-	-	-	-	-0.68 (0.020)

Numbers in parentheses are P-value. PDQ-39: Parkinson’s disease questionnaire with 39-items; SF-36: Short-form questionnaire with 36-items

## Discussion

In the current study, we evaluated responsiveness of Persian version of PDQ-39 questionnaire in patients with PD, who were under DBS surgery. Eleven patients were enrolled in the final analysis 6 months after DBS surgery. Responsiveness to change was perfect in all patients, and SRMs were higher than 0.70 for all domains of PDQ-39. Scores for all domains of PDQ-39 6 months after DBS surgery were decreased to improvement with statistically significant differences. These changes were compatible with H and Y score. All scales of SF-36 were improved 6 month after DBS surgery too. There was a fine correlation between SF-36 scales and relevant PDQ-39 6 months after surgery.

Mean age of patients was 51.8 years and the mean age at the onset of disease was 43 years. All patients just one were men. It has been shown epidemiologically that PD is more common in men than women.^22^

Nyholm et al. were evaluated the effect of duodenal levodopa infusion monotherapy versus oral polypharmacy in advanced PD.^23^ They used PDQ-39 for assessing QOL of patients with PD as an outcome measure. They showed QOL was improved. Although, their objective was not evaluation of responsiveness of PDQ-30, but changing in score of PDQ-39 was compatible with changing in clinical signs of patients. In our study, mean scores for all aspects of PDQ-39 were decreased significantly to improvement 6 months after DBS. Furthermore, clinical signs of patients with PD were improved during this period. It means, DBS surgery had a great effect on QOL of patients. Fitzpatric et al. also were assessed PDQ-39 responsiveness using SRM.^24^ They showed worsening of QOL of patients with PD after 4-month follow-up. They illustrated change in the PDQ-39 score was significantly correlated with self-reported change and change in SF-36. These findings are same with our study, and again show a good correlation between changing score of PDQ-39 with other measures and clinical changing. This finding indicates a good responsiveness and constructs validity of PDQ-39.

To determine the ability of PDQ-39 to change overtime, Harrison et al. used this measure and general health questionnaire-28 in patients with PD.^25^ They followed patients for 18 months. The PDQ-39 showed marked changes in levels of functioning. They concluded PDQ-39 is a sensitive device to monitor changes in patients with PD.

A systematic review was conducted to assess the effect of DBS on QOL of patients with PD. In this review, the authors showed a significant improvement in QOL (using PDQ-39) up to 6 months following DBS in three randomized controlled trials (RCTs) and in one nonrandomized trial and up to 12, 18, and 24 months postoperatively in one RCT.^26^ None of these trials aimed to assess responsiveness of PDQ-39 using SRM.

This study has some strength and limitation pints. The most important strength point was evaluation of responsiveness of PDQ-39 in patients with PD with DBS therapy. We evaluated responsiveness using SRM that is a standard method to assess responsiveness. This study is the first study with the aim of to determine responsiveness of Persian version of PDQ-39.

The most important limitation of the current study is low sample size. Although, with this sample size we could reach to our objectives. The main cause of this problem was the cost of DBS surgery for patients, and majority of this cost should pay out of pocket.

## Conclusion

We showed a Persian version of PDQ-39 has an acceptable responsiveness and could be used to assess as an outcome measure to evaluate the effect of therapies on PD. Further study with long term follow-up and larger sample is recommended to evaluate the consistency of these findings.

## Notes

**How to cite this article: **Shahidi GhA, Ghaempanah Z, Khalili Y, Nojomi M. Deep brain stimulation and responsiveness of the Persian version of Parkinson’s disease questionnaire with 39-items. Iran J Neurol 2014; 13(4): 220-5.
